# Supplementary material for: Rangeland dynamics: investigating vegetation composition and structure of urban and exurban prairie dog habitat
Source: PeerJ. 2015 Jan 29;3:e736. doi: 10.7717/peerj.736 (PMC4314087; doi:10.7717/peerj.736)
Supplement: Table S1 — (A) Repeated measures analyses of variance on absolute canopy cover of plant functional groups, litter and bare ground on- and off-colony (prairie dog) at urban and exurban sites in Fort Collins, Colorado, USA. Data were transformed using the arc-sine square root functions to meet assumptions of AOV. (B) Analyses of variance on absolute canopy cover of plant functional groups at peak standing crop on- and off-colony (prairie dog) at urban and exurban sites in Fort Collins, Colorado, USA. Data were transformed using the arc-sine square root functions to meet assumptions of AOV. [file peerj-03-736-s002.docx]

| **Table S1A. Repeated measures analyses of variance on absolute canopy cover of plant functional groups, litter and bare ground on- and off-colony (prairie dog) at urban and exurban sites in Fort Collins, Colorado, USA. Data were transformed using the arc-sine square root functions to meet assumptions of AOV.** | | | | |
| --- | --- | --- | --- | --- |
|  | **Num DF** | **Den DF** | **F Value** | **Pr > F** |
| **FORBS** |  |  |  |  |
| Site | 1 | 24 | 3.39 | 0.0781 |
| Colony | 1 | 24 | 63.04 | <.0001 |
| Site*Colony | 1 | 24 | 26.03 | <.0001 |
| Season | 2 | 24 | 1.74 | 0.1968 |
| Site*Season | 2 | 24 | 1.29 | 0.2928 |
| Colony*Season | 2 | 24 | 3.34 | 0.0526 |
| Site*Colony*Season | 2 | 24 | 2.47 | 0.1062 |
| **GRAMINOIDS** |  |  |  |  |
| Site | 1 | 24 | 94.64 | <.0001 |
| Colony | 1 | 24 | 135.72 | <.0001 |
| Site*Colony | 1 | 24 | 15.46 | 0.0006 |
| Season | 2 | 24 | 1.20 | 0.3187 |
| Site*Season | 2 | 24 | 0.93 | 0.4078 |
| Colony*Season | 2 | 24 | 0.44 | 0.6500 |
| Site*Colony*Season | 2 | 24 | 0.57 | 0.5744 |
| **SHRUBS** |  |  |  |  |
| Site | 1 | 24 | 45.04 | <.0001 |
| Colony | 1 | 24 | 88.78 | <.0001 |
| Site*Colony | 1 | 24 | 45.04 | <.0001 |
| Season | 2 | 24 | 0.16 | 0.8553 |
| Site*Season | 2 | 24 | 0.27 | 0.7656 |
| Colony*Season | 2 | 24 | 0.16 | 0.8553 |
| Site*Colony*Season | 2 | 24 | 0.27 | 0.7656 |
| **LITTER** |  |  |  |  |
| Site | 1 | 24 | 21.06 | 0.0001 |
| Colony | 1 | 24 | 9.11 | 0.0059 |
| Site*Colony | 1 | 24 | 3.07 | 0.0925 |
| Season | 2 | 24 | 2.54 | 0.0998 |
| Site*Season | 2 | 24 | 1.37 | 0.2721 |
| Colony*Season | 2 | 24 | 1.76 | 0.1934 |
| Site*Colony*Season | 2 | 24 | 0.26 | 0.7706 |
|  |  |  |  |  |
|  |  |  |  |  |
| **BARE GROUND** |  |  |  |  |
| Site | 1 | 24 | 48.37 | <.0001 |
| Colony | 1 | 24 | 47.39 | <.0001 |
| Site*Colony | 1 | 24 | 2.33 | 0.1401 |
| Season | 2 | 24 | 0.15 | 0.8631 |
| Site*Season | 2 | 24 | 0.04 | 0.9604 |
| Colony*Season | 2 | 24 | 1.42 | 0.2611 |
| Site*Colony*Season | 2 | 24 | 0.48 | 0.6266 |
|  |  |  |  |  |

|  |  |  |  |  |
| --- | --- | --- | --- | --- |
| **Table S1B. Analyses of variance on absolute canopy cover of plant functional groups at peak standing crop on- and off-colony (prairie dog) at urban and exurban sites in Fort Collins, Colorado, USA. Data were transformed using the arc-sine square root functions to meet assumptions of AOV.** | | | | |
|  | **Num DF** | **Den DF** | **F Value** | **Pr > F** |
| **PERENNIAL NATIVE WARM SEASON GRAMINOIDS** | | | | |
| Site | 1 | 8 | 0.79 | 0.3988 |
| Colony | 1 | 8 | 15.43 | 0.0044 |
| Site*Colony | 1 | 8 | 0.11 | 0.7525 |
| **PERENNIAL NATIVE COOL SEASON GRAMINOIDS** | | | | |
| Site | 1 | 8 | 25.48 | 0.0010 |
| Colony | 1 | 8 | 30.68 | 0.0005 |
| Site*Colony | 1 | 8 | 51.90 | <.0001 |
| **ANNUAL INTRODUCED COOL SEASON GRAMINOIDS** | | | | |
| Site | 1 | 8 | 10.32 | 0.0124 |
| Colony | 1 | 8 | 0.0139 | 0.9089 |
| Site*Colony | 1 | 8 | 0.2511 | 0.6298 |
| **PERENNIAL INTRODUCED COOL SEASON GRAMINOIDS** | | | | |
| Site | 1 | 8 | 131.29 | <.0001 |
| Colony | 1 | 8 | 212.83 | <.0001 |
| Site*Colony | 1 | 8 | 131.29 | <.0001 |
| **PERENNIAL NATIVE FORBS** | | | | |
| Site | 1 | 8 | 3.47 | 0.0996 |
| Colony | 1 | 8 | 1.01 | 0.3439 |
| Site*Colony | 1 | 8 | 0.39 | 0.5489 |
| **ANNUAL NATIVE FORBS** | | | | |
| Site | 1 | 8 | 33.19 | 0.0004 |
| Colony | 1 | 8 | 52.19 | <.0001 |
| Site*Colony | 1 | 8 | 16.80 | 0.0034 |
| **PERENNIAL INTRODUCED FORBS** | | | | |
| Site | 1 | 8 | 0.49 | 0.5024 |
| Colony | 1 | 8 | 34.73 | 0.0004 |
| Site*Colony | 1 | 8 | 11.40 | 0.0097 |
